# Liver and muscle glycogen oxidation and performance with dose variation of glucose–fructose ingestion during prolonged (3 h) exercise

**DOI:** 10.1007/s00421-019-04106-9

**Published:** 2019-03-06

**Authors:** Andy J. King, John P. O’Hara, Nicola C. Arjomandkhah, Josh Rowe, Douglas J. Morrison, Thomas Preston, Roderick F. G. J. King

**Affiliations:** 1 Carnegie School of Sport, Fairfax Hall, Research Institute for Sport, Physical Activity and Leisure, Leeds Beckett University, Leeds, LS6 3QT UK; 2 Scottish Universities Environmental Research Centre, Glasgow, UK; 3 Leeds Trinity University, Leeds, UK

**Keywords:** Muscle glycogen, Carbohydrate ingestion, Stable isotope, Exercise, Metabolism

## Abstract

**Purpose:**

This study investigated the effect of small manipulations in carbohydrate (CHO) dose on exogenous and endogenous (liver and muscle) fuel selection during exercise.

**Method:**

Eleven trained males cycled in a double-blind randomised order on 4 occasions at 60% $\dot {V}{{\text{O}}_{2\;\hbox{max} }}$ for 3 h, followed by a 30-min time-trial whilst ingesting either 80 g h^−1^ or 90 g h^−1^ or 100 g h^−1 13^C-glucose-^13^C-fructose [2:1] or placebo. CHO doses met, were marginally lower, or above previously reported intestinal saturation for glucose–fructose (90 g h^−1^). Indirect calorimetry and stable mass isotope [^13^C] techniques were utilised to determine fuel use.

**Result:**

Time-trial performance was 86.5 to 93%, ‘likely, probable’ improved with 90 g h^−1^ compared 80 and 100 g h^−1^. Exogenous CHO oxidation in the final hour was 9.8–10.0% higher with 100 g h^−1^ compared with 80 and 90 g h^−1^ (ES = 0.64–0.70, 95% CI 9.6, 1.4 to 17.7 and 8.2, 2.1 to 18.6). However, increasing CHO dose (100 g h^−1^) increased muscle glycogen use (101.6 ± 16.6 g, ES = 0.60, 16.1, 0.9 to 31.4) and its relative contribution to energy expenditure (5.6 ± 8.4%, ES = 0.72, 5.6, 1.5 to 9.8 g) compared with 90 g h^−1^. Absolute and relative muscle glycogen oxidation between 80 and 90 g h^−1^ were similar (ES = 0.23 and 0.38) though a small absolute (85.4 ± 29.3 g, 6.2, − 23.5 to 11.1) and relative (34.9 ± 9.1 g, − 3.5, − 9.6 to 2.6) reduction was seen in 90 g h^−1^ compared with 100 g h^−1^. Liver glycogen oxidation was not significantly different between conditions (ES < 0.42). Total fat oxidation during the 3-h ride was similar in CHO conditions (ES < 0.28) but suppressed compared with placebo (ES = 1.05–1.51).

**Conclusion:**

‘Overdosing’ intestinal transport for glucose–fructose appears to increase muscle glycogen reliance and negatively impact subsequent TT performance.

## Introduction

Fuel selection and energy expenditure during prolonged exercise are influenced by exercise intensity (Romijn et al. 1993) and exogenous substrate availability (Cox et al. 2016; King et al. 2018). The performance benefit of ingesting exogenous carbohydrate (CHO) is well documented (Currell and Jeukendrup 2008; McConell et al. 1999; Smith et al. 2010, 2013); however, the glycogen sparing capacity of exogenous CHO has received equivocal evidence (Newell et al. 2018) and may be dependent on the composition and dose of the ingested CHO. Furthermore, based on recent observations, the optimal CHO dose during exercise lasting more than 2–2.5 h may also be sensitive to ‘over-dosing’ the intestinal transport proteins for glucose and/or fructose is detrimental to substrate utilisation and exercise performance (King et al. 2018).

Exogenous CHO oxidation is increased when glucose–fructose formulations (relative to glucose only) are ingested at a rate of ~ 90 g h^−1^ during exercise (Jentjens et al. 2004a, b, 2006). Previously, maintaining high exogenous CHO oxidation rates was suggested as a possible mechanism behind the ergogenic effect of CHO ingestion during exercise (Jeukendrup 2008). But many studies investigating more than one CHO dose or formulation (e.g. glucose–fructose, glucose–sucrose mixtures) either did not measure exercise performance with possible beneficial exogenous CHO oxidation (Jentjens et al. 2004a, b, 2005) or exogenous oxidation with promising performance data (Baur et al. 2014; Smith et al. 2013; Tripplett et al. 2010).

The CHO dose may also play an important role and it remains to be seen if the CHO dose can be further optimised around the proposed upper limit for intestinal absorption of 90 g h^−1^ during endurance exercise of prolonged duration. Previously, we reported a dose effect of CHO ingestion on substrate utilisation during 2 h of high intensity (77% $\dot {V}{{\text{O}}_{2\;\hbox{max} }}$) cycling and performance in a 30-min time trial (King et al. 2018). Ingestion of a glucose–fructose solution (2:1) attenuated the rate of muscle glycogen oxidation with a small reduction in liver glycogen oxidation when the dose was sufficient (90 g h^−1^) to saturate the intestinal transport proteins, sodium-dependent glucose transporter 1 (SGLT1) and glucose transporter 5 (GLUT5). However, exceeding intestinal saturation rates for glucose–fructose increased the reliance on pre-existing stores of muscle glycogen, which had a detrimental effect on performance. In this respect, the 90 g h^−1^ dose of glucose–fructose was optimal for substrate utilisation and performance and agrees with previous performance (Smith et al. 2013) and hepatic glycogen oxidation data (Gonzalez et al. 2015). In contrast, the latter of these studies reported no change in muscle glycogen oxidation. This may in part be due to a methodological limitation in quantifying glycogen oxidation post-exercise with NMRS, but also attributable to the lower exercise intensity (50% *W*_peak_), where muscle glycogen may not be as crucial to prolonged exercise performance. Furthermore, the glucose–fructose dose used by Gonzalez et al. (2015) may have slightly exceeded intestinal transport, which may be detrimental to fuel utilisation and performance. It also remains to be seen if glucose:fructose doses can be optimised around the 90 g h^−1^ proposed upper limit and if any endogenous fuel utilisation effects remain at longer duration and lower intensity exercise.

As many endurance sports require performance in excess of 3 h, optimising fuelling strategies is a key concern for athletes and practitioners. However, empirical data on the role of multiple transportable CHO dose on endogenous fuel use in exercise lasting this long have not been researched. Therefore, this study sought to investigate if small alterations in ingested CHO dose affect liver and muscle glycogen utilisation during 3 h of prolonged exercise. It is hypothesised that exceeding the intestinal saturation rates for glucose and fructose will negatively affect substrate utilisation and exercise performance.

## Methods

### Participants

Eleven trained, healthy male cyclists volunteered to participate in this study. Participants were required to have trained for > 3 times per week in cycling-specific training for at least the last 2 years. The mean age, body mass, stature, $\dot {V}{{\text{O}}_{2\;\hbox{max} }}$, and maximal power output (*W*_max_) were 30.3 ± 6.5 years, 78.2 ± 10.5 kg, 179.6 ± 5.8 cm, 60.0 ± 4.3 ml kg^−1^ min^−1^, and 329.5 ± 33.2 W, respectively. Procedures and potential risks were explained before the study and all participants provided written informed consent. The study received institutional ethics approval conducted in accordance with the Declaration of Helsinki.

### Preliminary testing

Preliminary testing consisted of two parts: a maximal incremental cycle test to volitional exhaustion to determine *W*_max_ and $\dot {V}{{\text{O}}_{2\;\hbox{max} }}$, and a familiarisation effort for the 30-min time trial (TT) used to quantify exercise performance in the subsequent experimental trials. The objective of the TT was to complete the maximum amount of work possible within 30 min. This visit was conducted 1 week before the first experimental trial on an SRM high-performance ergometer (SRM, Germany) as described in detail elsewhere (King et al. 2018).

### Experimental design

Participants completed four experimental trials (each separated by 7 days) consisting of 180 min cycling at 60% $\dot {V}{{\text{O}}_{2\;\hbox{max} }}$, followed by a 30 min self-paced TT. The exercise intensity was chosen to represent a moderate intensity but glycogen demanding state (Van Loon et al. 2001) and the average energy demands of prolonged stage racing (Ebert et al. 2006). During each trial participants ingested 250 ml of one of four drinks solutions every fifteen minutes (starting at minute 15 into the exercise protocol). Three CHO solutions, each enriched with 150 mg per 75 g CHO of a universally labelled (U-^13^C_6_) glucose and fructose tracer (Sigma Aldrich, St Louis, MO) providing 80 g, 90 g and 100 g of glucose (D-glucose; Thornton and Ross Ltd, Huddersfield, UK) and fructose (Danisco, Kettering, UK) (glucose–fructose ratio 2:1) were prescribed in a randomised, double-blind design. A placebo trial (PLA) was also conducted to determine the background appearance of ^13^CO_2_ in expired air and the metabolic response without CHO ingestion. All formulations contained 26 mmol L^−1^ of NaCl (Saxa, Herts, UK), as well as artificial sweetener (aspartame, Morrisons’ plc, Bradford, UK) to blind the participants to each condition. The isotopic composition of the stock glucose and fructose was measured by isotope ratio mass spectrometry (IRMS (Isoprime, Cheadle, UK)), using L-fucose as an isotopic internal standard as previously described (Morrison et al. 2011) and determined to be − 25.68‰ and − 12.27‰ respectively. All ^13^C measurements are quoted with reference to the internationally accepted standard for carbon isotope measurements, Vienna Pee Dee Belemnite (VPDB). The final isotopic enrichment of the ingested CHO solutions was: 80 g h^−1^ = 146.20 ± 9.92‰, 90 g h^−1^ = 149.18 ± 3.19‰ and 100 g h^−1^ = 145.70 ± 6.97‰. All ^13^C measurements are quoted with reference to the internationally accepted standard for carbon isotope measurements, Vienna Pee Dee Belemnite (VPDB).

### Diet and physical activity before testing

Participants recorded their food intake and physical activity during the 48 h before the first experimental trial and were instructed to repeat the same diet and activity pattern in the 48 h before subsequent trials. In the 24 h before each experimental trial, participants were required to not undertake any strenuous physical activity and avoid alcohol and caffeine consumption. Further, participants were also asked to undergo an intense training session 48 h before each visit to deplete glycogen stores, reducing background levels of ^13^Carbon (Harvey et al. 2007). Throughout the experimental trials, participants were asked to refrain from ingesting foods with a high natural ^13^C:^12^C abundance (i.e. plants with a C4 photosynthetic cycle, or animals fed with such plants). Each participant was provided with a list of foods to avoid (Morrison et al. 2000). This precaution ensures that background ^13^C enrichment of expired CO_2_ from endogenous substrate stores is less likely to be affected by unintentional but natural fluctuations of dietary ^13^C. Before each test, a standardised evening meal was consumed 10–12 h before arrival at the laboratory (total, 1443 kcal; 53% CHO, 17% fat, and 30% protein) and participants were instructed to consume 500 ml of water on the morning of each trial.

### Experimental trials

After a 10–12 h overnight fast, participants reported to the laboratory on each occasion between 0700 h and 0900 h. Upon arrival at the laboratory, an in dwelling catheter (20 gauge Introcan Safety®, B. Braun Medical Ltd., Sheffield, UK) was inserted into an antecubital vein for regular blood sampling. Over the next 10 min resting $\dot {V}{{\text{O}}_2}$ and $\dot {V}{\text{C}}{{\text{O}}_{\text{2}}}$ measurements were made with participants sitting on the cycle ergometer using an online gas analysis system (Metalyser, Cortex, Germany), which was calibrated following the manufacturer’s instructions. For the measurement of ^13^CO_2_:^12^CO_2_ in expired air at rest, 12 ml Exetainers (SerCon Ltd, Crewe, UK) of expired gas were collected in duplicate via a mixing chamber (Jaeger, Germany).

Participants then completed 180 min of cycling at 60% $\dot {V}{{\text{O}}_{2\;\hbox{max} }}$ on a high-performance ergometer. $\dot {V}{{\text{O}}_2}$_,_$\dot {V}{\text{C}}{{\text{O}}_{\text{2}}}$ and heart rate (HR) were measured for 5 min every 15 min until the end of exercise. Samples of expired gas for ^13^CO_2_:^12^CO_2_ analysis were collected during the final 60 s of each 15-min period. Samples for the analysis of plasma glucose, plasma lactate, serum insulin, serum-free fatty acids were drawn every 15 min and for ^13^C plasma glucose enrichment at 60 min and every 30 min thereafter. Following each completed 15-min period of data collection, one of the 250 ml drink solutions was given to the participants, who were instructed to consume the drink as quickly as comfortably possible.

### Analyses

Aliquots of plasma and serum prepared by centrifugation were analysed for selected metabolites. Glucose (glucose oxidase kit; Instrumentation Laboratory, Monza, Italy, Inter assay CV: 4.9%, Intra assay CV: 2.3%) and lactate (Lactate kit, Randox, County Antrim, UK, Inter CV: 4.5%, Intra CV: 2.7%) were analysed by spectrophotometry (iLab 300 plus, ILab, UK). Insulin was analysed using a chemoiluminometric immunoassay (ADIVA Centaur, Bayer diagnostics, Berkshire, UK, Inter CV: 3.2–4.6%, Intra CV: 2.6–5.9%). Non-esterified free fatty acid content of serum was analysed by an acyl-CoA synthetase and oxidase assay (NEFA-HR2, Wako Chemicals GmbH, Germany, Inter assay CV: 1.5%).

The ^13^CO_2_:^12^CO_2_ in expired air was determined by IRMS. The isotopic ratio (^13^C:^12^C) is derived against laboratory CO_2_ (itself calibrated against VPDB) from the ion beam area ratio measurements with correction of the small contribution of ^12^C^16^O^17^O at m/z 45 [Craig correction; (Craig 1957)]. The ^13^C:^12^C in plasma glucose was determined using LC-IRMS as described in detail previously (Morrison et al. 2011). Plasma samples were prepared by ultrafiltration (30,000 molecular weight cutoff tubes, Amicon Ultra 4, Millipore, Watford, UK), with an internal standard added (L-fucose, C_6_H_12_O_5_, Sigma-Aldrich) and separated by liquid chromatography to separate the glucose from other constituents prior to “wet-oxidation” and IRMS analysis of the resulting CO_2_.

### Calculations

Total CHO and fat oxidation (g.min^−1^) were computed from $\dot {V}{{\text{O}}_2}$ and $\dot {V}{\text{C}}{{\text{O}}_{\text{2}}}$ (L.min^−1^) using the stoichiometric equations of Frayn (Frayn 1983), with protein oxidation during exercise assumed to be negligible.

1 $${\text{CHO }}={\text{ }}\left( {{\text{4}}.{\text{55}} \times \dot {V}{\text{C}}{{\text{O}}_{\text{2}}}} \right) - \left( {{\text{3}}.{\text{21}} \times \dot {V}{{\text{O}}_{\text{2}}}} \right)$$

2 $${\text{Fat }}={\text{ }}\left( {{\text{1}}.{\text{67}} \times \dot {V}{{\text{O}}_{\text{2}}}} \right) - \left( {{\text{1}}.{\text{67}} \times \dot {V}{\text{C}}{{\text{O}}_{\text{2}}}} \right)$$

The isotopic enrichment of the ingested glucose and fructose, (*R*_exo_), and expired air (*R*_exp_) was expressed in standard δ^13^C units (‰) relative to VPDB (Craig 1953). Exogenous glucose oxidation derived from glucose and the combined ingestion of glucose and fructose (CHO_EX)_ was computed using the following equation (Peronnet et al. 1990), with the placebo condition establishing the background ^13^CO_2_:^12^CO_2_ during exercise.

3 $${\text{CH}}{{\text{O}}_{{\text{EX}}}}\left( {g{{\hbox{min} }^{ - 1}}} \right){\text{ }}={\text{ }}\dot {V}{\text{C}}{{\text{O}}_2}\left[ {\left( {{R_{\exp }}-{R_{{\text{ref}}}}} \right)/\left( {{R_{{\text{exo}}}}-{R_{{\text{ref}}}}} \right)} \right]/k$$

where $\dot {V}{\text{C}}{{\text{O}}_{\text{2}}}$ is in L.min^−1^, *R*_exp_ is the isotopic composition of expired CO_2_, *R*_ref_ is the isotopic composition of expired CO_2_ at the same time point with ingestion of placebo, *R*_exo_ is the isotopic composition of the ingested solution and *k* (0.747 L g^−1^) is the volume of CO_2_ provided by the complete oxidation of glucose.

Computations were made on the assumption that, in response to exercise, ^13^C is not irreversibly lost in pools of tricarboxylic acid cycle intermediates and/or bicarbonate and that ^13^CO_2_ recovery in expired gases was complete or almost complete during exercise (Trimmer et al. 2001). Such computation has been shown to underestimate exogenous oxidation rates at the beginning of exercise because of the delay between ^13^CO_2_ production in tissues and its exhalation (Pallikarakis et al. 1991). Therefore, carbohydrate oxidation data are presented for the second and third hours of the 3-h protocol to allow for a steady-state condition of ^13^C in the bicarbonate pool to be reached (Robert et al. 1987).

Based on the ^13^C isotopic composition of plasma glucose (*R*_glu_), the oxidation rate of plasma CHO was calculated (Peronnet et al. 1998):

4 $$\begin{aligned}&{\text{Plasma CHO }}\left( {g{{\hbox{min} }^{ - 1}}} \right)\\&=\dot {V}{\text{C}}{{\text{O}}_2}\left[ {\left( {{R_{{\text{exp}}}}-{R_{{\text{ref}}}}} \right)/\left( {{R_{{\text{glu}}}}-{R_{{\text{ref}}}}} \right)} \right]/k \end{aligned}$$

Endogenous CHO oxidation was calculated as the differences between total CHO oxidation and exogenous CHO oxidation. The oxidation rate of muscle glycogen (g min^−1^), either directly or through the lactate shuttle (Brooks 1986), was calculated by subtracting plasma glucose oxidation from total carbohydrate oxidation (Eq. 5). Finally, the amount of glucose released from the liver was estimated as the difference between plasma glucose and exogenous carbohydrate oxidation (Eq. 6) (Peronnet et al. 1998):

5 $$\begin{aligned}&{\text{Muscle oxidation }}\\ &={\text{ total CHO oxidation }}-{\text{ plasma glucose CHO oxidation,}}\end{aligned}$$

6 $$\begin{aligned}&{\text{Liver oxidation }}\\ &={\text{ plasma glucose CHO oxidation }}-{\text{ exogenous oxidation,}}\end{aligned}$$

### Statistical analyses

The mean value observed for a given variable is presented with the associated standard deviation (mean ± SD) and where comparisons between conditions made, as the mean difference with associated confidence limits at the 95% level (mean, 95% CI range) with Cohen’s d effect size [e.g. mean difference, lower limit to upper limit (ES)] as recommended by Hopkins et al. (2009).

To provide meaningful terms to the effectiveness of CHO ingestion on exercise performance, a probabilistic magnitude-based inference analysis was conducted to analyse the effect of CHO ingestion on the mean power output during the 30-min TT. Using the coefficient of variation (2.4%) of laboratory cycling TT performance (Hopkins et al. 1999) and the smallest worthwhile change in athletic performance (0.5 × CV), the smallest meaningful effect in power output between conditions was computed to be 1.2%. The effect of CHO ingestion was expressed as a percentage change relative to placebo ingestion following back transformation of the mean of the natural logarithm of the power outputs. The chance that the true value of the effect was larger than the smallest meaningful effect on the 30 min TT was computed and qualitative terms assigned (Hopkins et al. 2009): < 1%, almost certainly not; < 5%, very unlikely; < 25%, unlikely or probably not; < 50%, possibly not; > 50%, possibly; > 75%, likely or probable; > 95%, very likely; > 99% almost certain. For non-performance variables (heart rate, *V*O_2_, substrate oxidation, and plasma glucose and lactate, serum-free fatty acid and insulin concentrations) where a smallest worthwhile change is difficult to calculate, statistical comparisons were also made using a one-way (dose) or two-way (dose x time) repeated measures ANOVA with Bonferroni post hoc adjustment (SPSS 20, IBM, New York, USA) as well as Cohen’s d effect sizes *(ES). ES threshold values were set as 0.2. 0.6, 1.2, 2.0, and 4.0 for small, moderate, large, very large, and extremely large effects, respectively (Hopkins et al. 2009).

## Results

### $\dot {V}{{\text{O}}_2}$, $\dot {V}{\text{C}}{{\text{O}}_{\text{2}}}$ and heart rate

$\dot {V}{{\text{O}}_2}$ and $\dot {V}{\text{C}}{{\text{O}}_{\text{2}}}$ increased by a small but significant amount over time, i.e. between the 1st and 2nd hours (pooled data; ES = 0.19, *P* = 0.01 and ES = 0.01, *P* = 0.01, respectively, Table 1) and the 2nd and 3rd hours (pooled data; ES = 0.20, *P* = 0.01 and ES = 0.15, *P* = 0.014, respectively). There were small but significant differences in $\dot {V}{\text{C}}{{\text{O}}_{\text{2}}}$ between 100 g h^−1^ and PLA during the 2nd and 3rd hours of the 3-h ride (ES = 1.36 and 1.99, *P* = 0.02 and 0.01, see Table 1 for comparisons); however, all other comparisons for $\dot {V}{{\text{O}}_2}$ and $\dot {V}{\text{C}}{{\text{O}}_{\text{2}}}$ were not significantly different, producing small effects (ES < 0.55, *P* > 0.62). RER between CHO conditions was not different throughout the 3-h ride (ES = 0.02 to 0.22, *P* > 0.9), but was higher in all CHO conditions in the 2nd and 3rd h relative to PLA (large ES > 1.41).

**Table 1:** Respiratory gas exchange, heart rate and substrate utilisation over the 1st, 2nd 3rd hours of the 3-h ride

	Condition				
	Hour	Pla	80 g h^−1^	^−1^90 g h	100 g h^−1^
HR (b min^−1^)	1st	136 ± 13	135 ± 12	136 ± 6	138 ± 12
	2nd	140 ± 14	139 ± 13	140 ± 13	142 ± 13
	3rd	145 ± 14	141 ± 13	144 ± 14	144 ± 13
*V*O_2_ (L min^−1^)	1st	2.91 ± 0.31	3.06 ± 0.46	2.87 ± 0.44	3.11 ± 0.36
	2nd	3.03 ± 0.30	3.01 ± 0.49	3.00 ± 0.47	3.22 ± 0.34
	3rd	3.12 ± 0.37	3.10 ± 0.49	3.13 ± 0.45	3.25 ± 0.42
*V*CO_2_ (L min^−1^)	1st	2.60 ± 0.27	2.77 ± 0.38	2.62 ± 0.36	2.84 ± 0.30
	2nd	2.66 ± 0.27	2.78 ± 0.44	2.76 ± 0.42	3.00 ± 0.30
	3rd	2.69 ± 0.30	2.87 ± 0.44	2.86 ± 0.41	3.02 ± 0.38
RER	1st	0.89 ± 0.03	0.91 ± 0.03	0.92 ± 0.03	0.91 ± 0.03
	2nd	0.88 ± 0.03	0.92 ± 0.03	0.92 ± 0.03	0.93 ± 0.03
	3rd	0.86 ± 0.03	0.92 ± 0.03	0.92 ± 0.03	0.93 ± 0.02
CHO_ox_ (g)	1st	149.6 ± 22.7	162.4 ± 28.7	166.6 ± 27.3	167.5 ± 18.3
	2nd	144.0 ± 25.9	177.4 ± 33.0	182.5 ± 34.4	189.9 ± 19.6
	3rd	137.5 ± 25.3	179.0 ± 32.6	185.4 ± 37.3	192.3 ± 19.8
Fat_ox_ (g)	1st	33.6 ± 10.0	30.1 ± 11.8	25.6 ± 9.2	28.8 ± 10.8
	2nd	36.4 ± 9.2	24.0 ± 10.5	21.4 ± 8.8	23.4 ± 11.0
	3rd	40.3 ± 10.3	24.1 ± 10.6	23.3 ± 8.6	23.8 ± 11.0

Data are heart rate in b min^−1^, $\dot {V}{{\text{O}}_2}$, $\dot {V}{\text{C}}{{\text{O}}_{\text{2}}}$ in L min^−1^, respiratory exchange ratio (RER), and CHO and fat oxidation in grams. The 1st hour is presented in the top line of each variable, and the 2nd hour in the middle line and the 3rd hour in the bottom line. All values are mean ± SD. a denotes PLA significantly lower than HGF

HR did not significantly increase between the 1st and 2nd hour or 2nd and 3rd hour of the constant load ride in any condition (main effect ES = 0.30, *P* = 0.19), Table 1. There were no differences between conditions during the 3-h ride (small effects, ES < 0.25, *P* = 1.00).

### Total carbohydrate and fat oxidation

Total energy expenditure was not significantly different between conditions for the 3 h of continuous cycling (PLA = 2803.9 ± 278.2 kCal, 80 g h^−1^ = 2874.0 ± 448.2 kCal, 90 g h^−1^ = 2878.6 ± 434.7 kCal, 100 g h^−1^ = 2978.3 ± 288.7.7 kCal, ES < 0.60, *P* > 0.18) or during the 3rd hour (PLA = 957.5 ± 110.8 kCal, 80 g h^−1^ = 978.8 ± 157.6 kCal, 90 g h^−1^ = 1000.1 ± 157.0 kCal, 100 g h^−1^ = 1016.6 ± 112.5 kCal, ES < 0.53, *P* > 0.16). In addition, no effects were seen between the 1st and 2nd hour or 2nd and 3rd hour of exercise (Table 1; ES < 0.44, *P* > 0.25).

Absolute CHO oxidation during the 3-h ride was lower with ingestion of PLA relative to 80 g h^−1^ and 90 g h^−1^, a large but non-significant effect (ES = 1.20 and 1.36, *P* = 0.09 and 0.09), and significantly lower than 100 g h^−1^ (very large effect, ES = 2.08, *P* = 0.002, see Table 1 for data). Absolute CHO oxidation followed the same pattern in the 3rd h of the 3-h ride; however, all effects were larger (ES = 1.40–2.35), with a significant difference between PLA and 100 g h^−1^ (*P* = 0.01). Differences between 80 g h^−1^ and 90 g h^−1^ and PLA remained non-significant (*P* = 0.06 and 0.064 respectively). In contrast, fat oxidation was highest with PLA ingestion producing a moderate to large effect for 3-h ride, (ES = 1.05–1.51, *P* = 0.02 vs. 90 g h^−1^ and 0.04 vs. 100 g h^−1^). However, in the 3rd hour, PLA fat oxidation was significantly higher (*P* = 0.008–0.02, large effects, ES = 1.55 to 1.80). There were no differences between CHO conditions for total CHO or fat oxidation across the 3-h (ES < 0.42, *P* = 1.00) or the 3rd hour (ES < 0.49, *P* = 1.00).

In addition, the relative contribution of CHO to energy expenditure was higher with CHO ingestion (range 73. 7 ± 9.9–76.1 ± 8.4%) than PLA (61.7 ± 9.1%) for both the whole 3-h ride (large ES = 1.26–1.71, *P* = 0.021–0.05) and the 3rd h (large ES = 1.53–2.19, *P* = 0.005–0.04, Fig. 2). The lower relative CHO oxidation in PLA was associated with a concomitant large (and significant) increase in the relative contribution of fat to the energy yield compared with CHO ingestion (effect sizes and P values the same as reported for CHO). Additionally, there were no significant differences between CHO conditions for the relative contribution of CHO and fat either during the whole 3-h ride (ES = 0.06–0.27, *P* = 1.00) or the 3rd hour (ES = 0.32–0.70, *P* = 0.24–1.00). However, the difference in the relative contribution of CHO and fat in 90 g h^−1^ produced a moderate effect to 100 g h^−1^ (6.2% mean difference, *P* = 0.24).

### δ ^13^CO_2_ in expired gas and δ ^13^C in plasma glucose

**Fig. 1:**
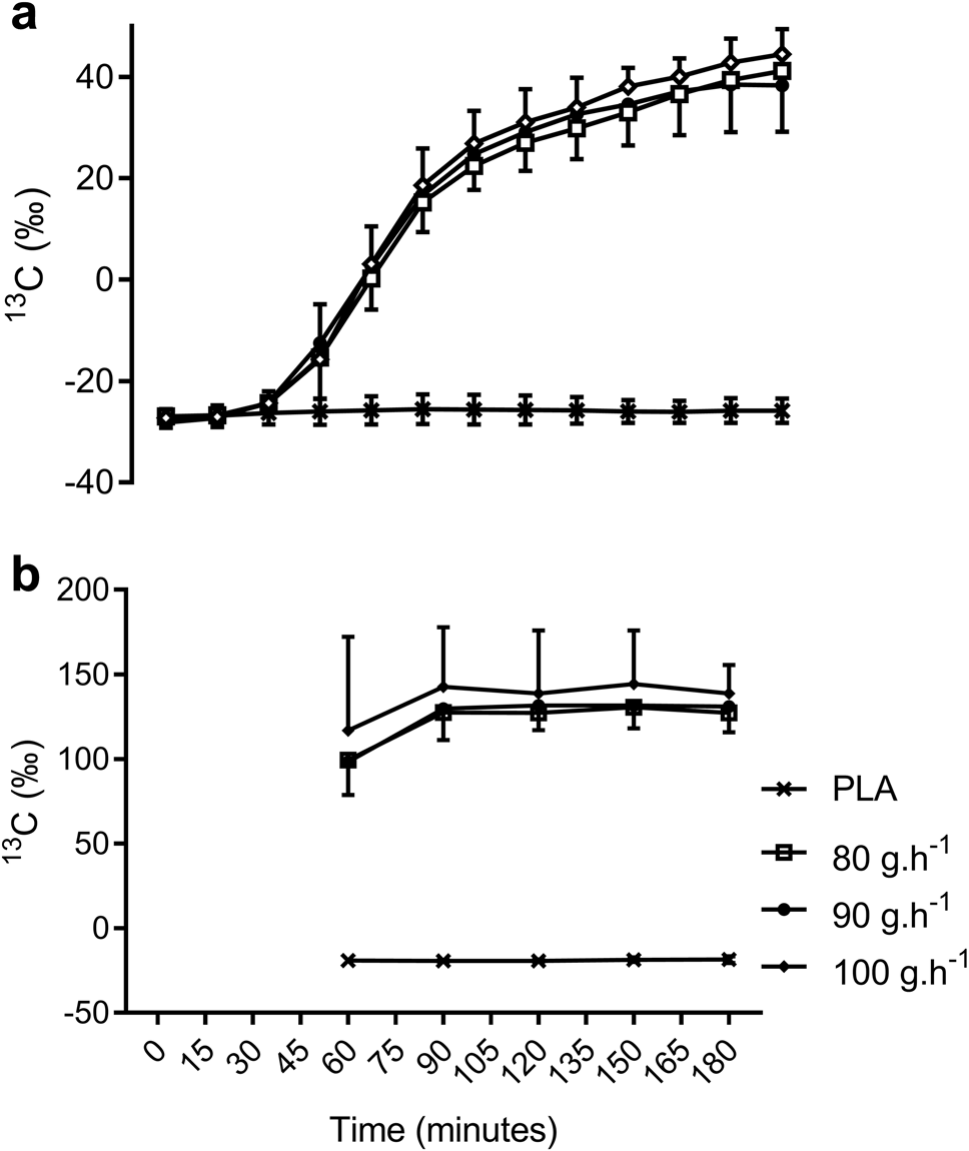
**a**^13^CO_2_:^12^CO_2_ (δ^13^C) in expired air over the 3-h ride and **b**^13^C:^12^C in plasma glucose during the second hour of the 3-h ride. See text for statistical and ES comparisons

The δ ^13^CO_2_ in expired gas was similar between all conditions at rest before exercise (ES = 0.23–0.42, *P* = 0.37–1.00). In PLA, the δ ^13^CO_2_ in expired gas increased over time by 1.4‰ (ES = 0.73) from the start to the end of exercise. These data were used as the background correction for the calculation of exogenous CHO and plasma glucose oxidation for each CHO condition. The δ ^13^CO_2_ in expired gas significantly increased over time from the start of exercise following the ingestion of all three ^13^C enriched CHO solutions (Fig. 1a). Each condition reached maximal values at 180 min; however, no significant differences were observed between CHO conditions at any time point, despite a moderately lower δ ^13^CO_2_ in 80 g h^−1^ relative to 90 g h^−1^ and 100 g h^−1^ at 180 min.

The isotopic composition of plasma glucose (δ ^13^C) increased by 0.5‰ from 60 to 180 min of exercise with ingestion of PLA (ES = 0.32, *P* = 1.00, Fig. 1b). In all CHO conditions, there was a significant rise in plasma glucose δ ^13^C between 60 and 90 min (ES = 0.43–1.17, *P* = 0.002–0.05) which remained similar for all conditions between 90 and 180 min (ES = 0.07–0.35, *P* > 0.11). The isotopic composition of plasma glucose was highest for 100 g h^−1^ during the last two hours of exercise, with a small effect (ES = 0.47–0.54) being relative to the other CHO conditions until 180 min where a moderate effect was observed between 100 g h^−1^ and 80 g h^−1^ (ES = 0.74, *P* = 0.20).

### Sources of oxidised glucose (exogenous and endogenous carbohydrate)

**Fig. 2:**
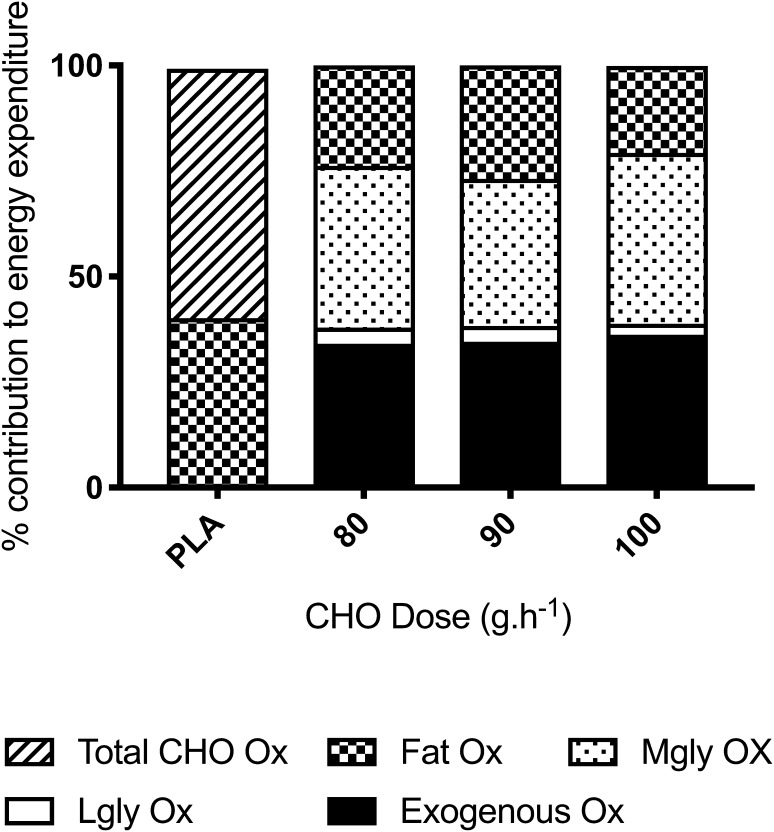
Percentage energy contributions from various substrates during the hour 2 of the 3-h ride. See text for statistical and ES comparisons. Mgly – muscle glycogen oxidation, *Lgly* liver glycogen oxidation

**Fig. 3:**
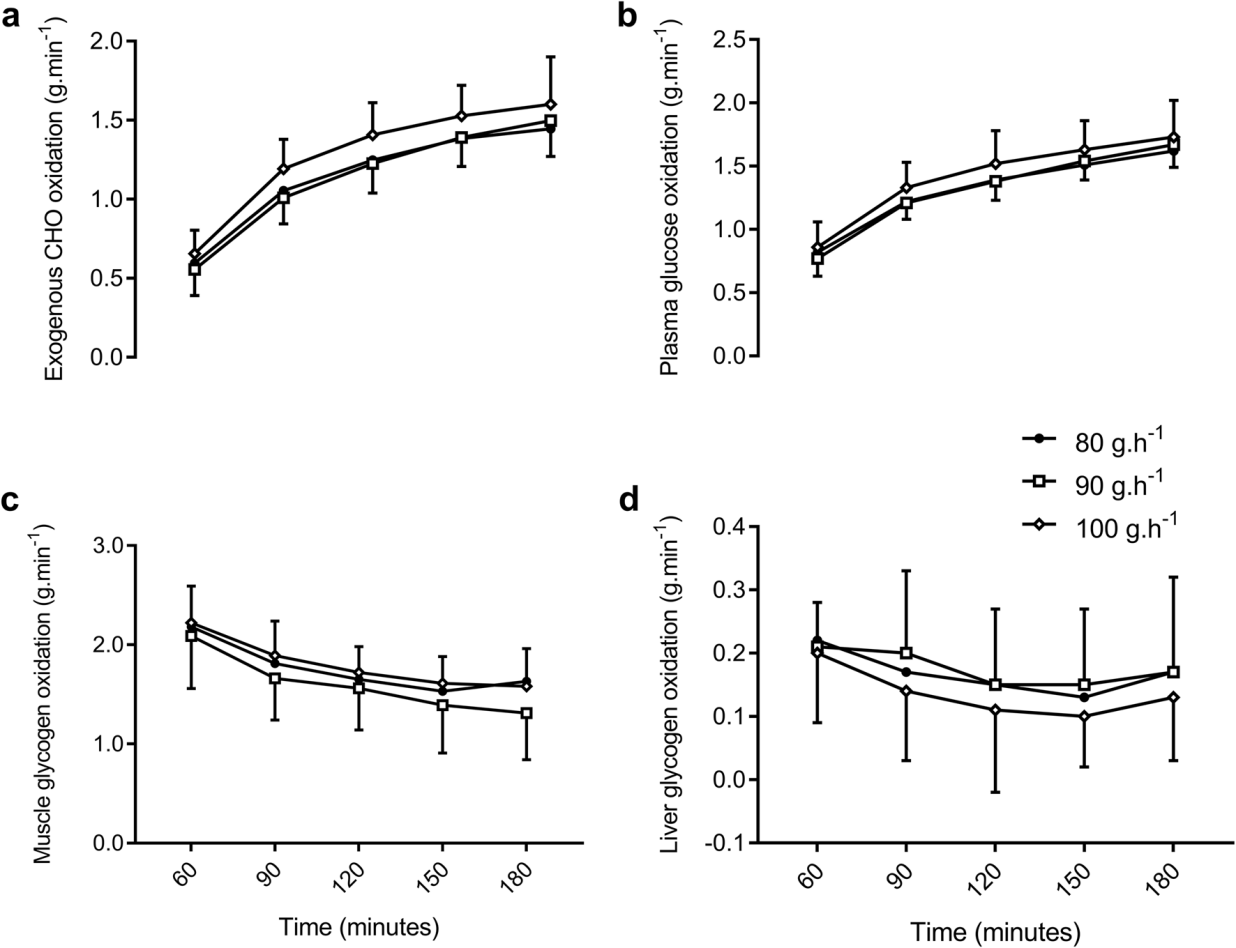
Sources of oxidised glucose and muscle glycogen during hours 2 and 3 of the 3-h ride. See text for statistical and ES comparisons

During the final hour of exercise, the rate of exogenous CHO oxidation was highest at the end of exercise (180 min) in all conditions with a small difference between 100 g h^−1^ and 80 g h^−1^ and 90 g h^−1^ (ES = 0.39 and 0.55, *P* = 0.28 and 1.00 respectively, Fig. 3a). There were no significant or moderate or large effects observed at all other time points between conditions during the 3-h ride. The absolute oxidation of exogenous CHO was moderately higher (8.1 and 9.6 g, ES = 0.64 and 0.96) in 100 g h^−1^ than 80 g h^−1^ and 90 g h^−1^ respectively, but was not significantly different between CHO conditions in the 2nd hour (*P* = 0.07–1.00). A similar magnitude of difference between conditions was seen in the final hour, but no significant differences were detected in the absolute oxidation of exogenous CHO, despite a moderate effect (ES = 0.64 and 0.70) between 100 g h^−1^ and 80 g h^−1^ and 90 g h^−1^ respectively (*P* = 0.11–1.00). The relative contribution of exogenous CHO to total energy expenditure was not significantly different in the 2nd hour (range 25.6 ± 4.6–27.5 ± 4.2, ES < 0.44, *P* > 0.33) or the 3rd hour (ES < 0.56, *P* > 0.28, Fig. 2). The relative contribution of exogenous CHO was significantly greater (large effect size) in the final hour than the 2nd hour (ES > 1.78, *P* < 0.01).

Compared with PLA, CHO ingestion caused a moderate reduction in the absolute amount of endogenous CHO oxidised in the 3rd hour of exercise with 80 g h^−1^ (30.4, 8.3 to 52.6 g, *P* = 0.13, ES = 1.12) and 100 g h^−1^ (23.1, 5.3 to 40.9, *P* = 0.35, ES = 0.99) and a large reduction with 90 g h^−1^ (36.6, 14.4 to 58.8, *P* = 0.13, ES = 1.22, Table 2). During the 2nd hour, all differences to PLA were moderate (ES = 0.68–0.97) but non-significant, (*P* > 0.37). Differences in absolute endogenous CHO oxidation between CHO conditions were not significantly modified during the 3rd hour of exercise. With 90 g h^−1^, a small reduction was seen relative to 80 g h^−1^ (− 6.2, − 22.0 to 9.7 g, *P* = 1.00. ES = 0.21) and 100 g h^−1^ (− 13.5, − 29.4 to 2.3, *P* = 0.75, ES = 0.51).

**Table 2:** Comparisons of CHO oxidation source during the third hour

	CHO Ox (g)	Difference in CHO oxidation (g)	
		Low	Med
Exogenous CHO			
80 g h^−1^	81.6 ± 17.0		
90 g h^−1^	82.9 ± 11.2	1.3, − 9.4 to 12.1 ES = 0.09, *P* = 1.00	
100 g h^−1^	91.1 ± 12.3	9.6, 1.4 to 17.7 ES = 0.64, *P* = 0.10	8.2, 2.1 to 18.6 ES = 0.70, *P* = 0.39
Endogenous CHO			
80 g h^−1^	100.7 ± 27.1		
90 g h^−1^	94.5 ± 32.6	6.2, − 22.8 to 10.5 ES = 0.21, P = 1.00	
100 g h^−1^	108.0 ± 18.8	7.3, − 9.0 to 23.8 ES = 0.32, P = 1.00	13.5, − 3.1 to 30.2 ES = 0.51, P = 0.37
Plasma glucose			
80 g h^−1^	90.6 ± 15.0		
90 g h^−1^	92.0 ± 8.5	1.4, − 6.4 to 9.1 ES = 0.11, *P* = 1.00	
100 g h^−1^	97.6 ± 14.6	7.0, − 1.3 to 15.3 ES = 0.47, *P* = 0.34	5.6, − 3.3 to 14.5 ES = 0.47, *P* = 0.67
Liver glycogen			
80 g h^−1^	9.0 ± 7.6		
90 g h^−1^	9.1 ± 7.1	0.1, − 4.3 to 4.3 ES = 0.00, *P* = 1.00	
100 g h^−1^	6.4 ± 5.2	− 2.6 − 6.8 to 1.6 ES = 0.40, *P* = 0.70	− 2.6, − 5.6 to 0.4 ES = 0.42, *P* = 0.31
Muscle glycogen			
80 g h^−1^	91.6 ± 24.6		
90 g h^−1^	85.4 ± 29.3	6.2, − 23.5 to 11.1 ES = 0.23, *P* = 1.00	
100 g h^−1^	101.6 ± 16.6	10.0, − 5.1 to 25.0 ES = 0.47, *P* = 0.61	16.1, 0.9 to 31.4 ES = 0.68, *P* = 0.16

Values given are comparisons of CHO oxidation from various sources over the third hour of the 3 h ride between 80 g h^−1^, 90 g h^−1^ and 100 g h^−1^ glucose: fructose ingestion. [1st line: mean ± s.d., absolute difference between conditions with associated 95% confidence intervals; 2nd line: Cohen’s d effect size and *P* value (ANOVA with Bonferroni post hoc comparison)]

With 100 g h^−1^, plasma glucose oxidation rates increased more quickly, producing moderate, but non-significant differences to 80 and 90 g h^−1^ at 90 and 120 min (ES = 0.72 and 0.68, *P* = 0.91 and 0.93). At 150 and 180 min, the rate of plasma glucose oxidation remained slightly higher with 100 g h^−1^, but differences to 80 and 90 g h^−1^ became small (150 min, ES = 0.42 and 0.46, *P* = 1.00; 180 min, ES = 0.43 and 0.28, *P* = 1.00). Absolute plasma glucose oxidation in the 3rd hour was not significantly different between conditions, with all differences producing small effects (ES = 0.00 to 0.42, *P* = 0.34–1.00, Table 2).

Similarly, rates of liver-derived glucose oxidation (Fig. 3d) were not significantly different between CHO conditions. When the CHO dose was increased from 80 to 90 g h^−1^, effects did not exceed > 0.26 at any time point in the last 2 h of exercise (*P* = 1.00). With 100 g h^−1^, a small reduction was observed (ES = 0.22–0.53, *P* = 0.34–0.80). During the 3rd hour of exercise, the total amount of glucose released from the liver was the same between 80 and 90 g h^−1^ (9.0 vs. 9.1 g) but slightly higher with 100 g h^−1^ (ES = 0.40 and 0.42 respectively, *P* = 0.70 and 0.31). Expressed as the relative contribution to total energy expenditure during the 3rd hour, these effects remained small with 100 g h^−1^ providing a greater contribution than 80 g h^−1^ (1.3, − 0.4 to 3.0%, ES = 0.51, *P* = 0.30) and 90 g h^−1^ (1.2, − 0.1 to 2.4, ES = 0.47, *P* = 0.56, Fig. 2).

Rates of muscle glycogen oxidation were not different between conditions at 60 min (ES < 0.28, *P* = 1.00, Fig. 3c) but became slightly attenuated with 90 g h^−1^ relative to 100 g h^−1^ from 75 min onwards. Throughout the 2nd and 3rd hours of the 3-h ride, differences in the rate of muscle glycogen oxidation between 80 and 90 g h^−1^ were not significantly different (ES < 0.31, *P* > 0.84) except at 180 min, where the difference was greatest, producing a moderately lower rate with 90 g h^−1^ (0.32, 0.00 to 0.60 g.min^−1^, ES = 0.66, *P* = 0.12). Throughout the 2nd and 3rd h, the rate of muscle glycogen oxidation remained moderately lower with 90 g h^−1^ than 100 g h^−1^ with the greatest difference also seen at 180 min, though effects were not significantly different (0.27, 0.01 to 0.53, ES = 0.65, *P* = 0.20). With 100 g h^−1^, the absolute amount of muscle glycogen oxidised in the final hour of exercise was higher than 80 g h^−1^ (10.0, − 5.1 to 25.0 g, ES = 0.47, *P* = 0.61) and 90 g h^−1^ (16.1, 0.9 to 31.4 g, ES = 0.68, *P* = 0.16). The difference between 80 and 90 g h^−1^ was small (− 6.2, − 23.5 to 11.1 g, ES = 0.23, P = 1.00). In terms of the relative contribution to total energy expenditure, the effect of CHO on muscle glycogen oxidation when the dose was increased from 90 to 100 g h^−1^ was also moderate (5.6, 0.9 to 10.3%, ES = 0.72, *P* = 0.12, Fig. 2). The difference between 80 and 90 g h^−1^, though still lower with 90 g h^−1^ was small (− 3.5, − 10.8 to 3.7%, ES = 0.36, *P* = 1.00).

### Circulatory metabolites and insulin

**Fig. 4:**
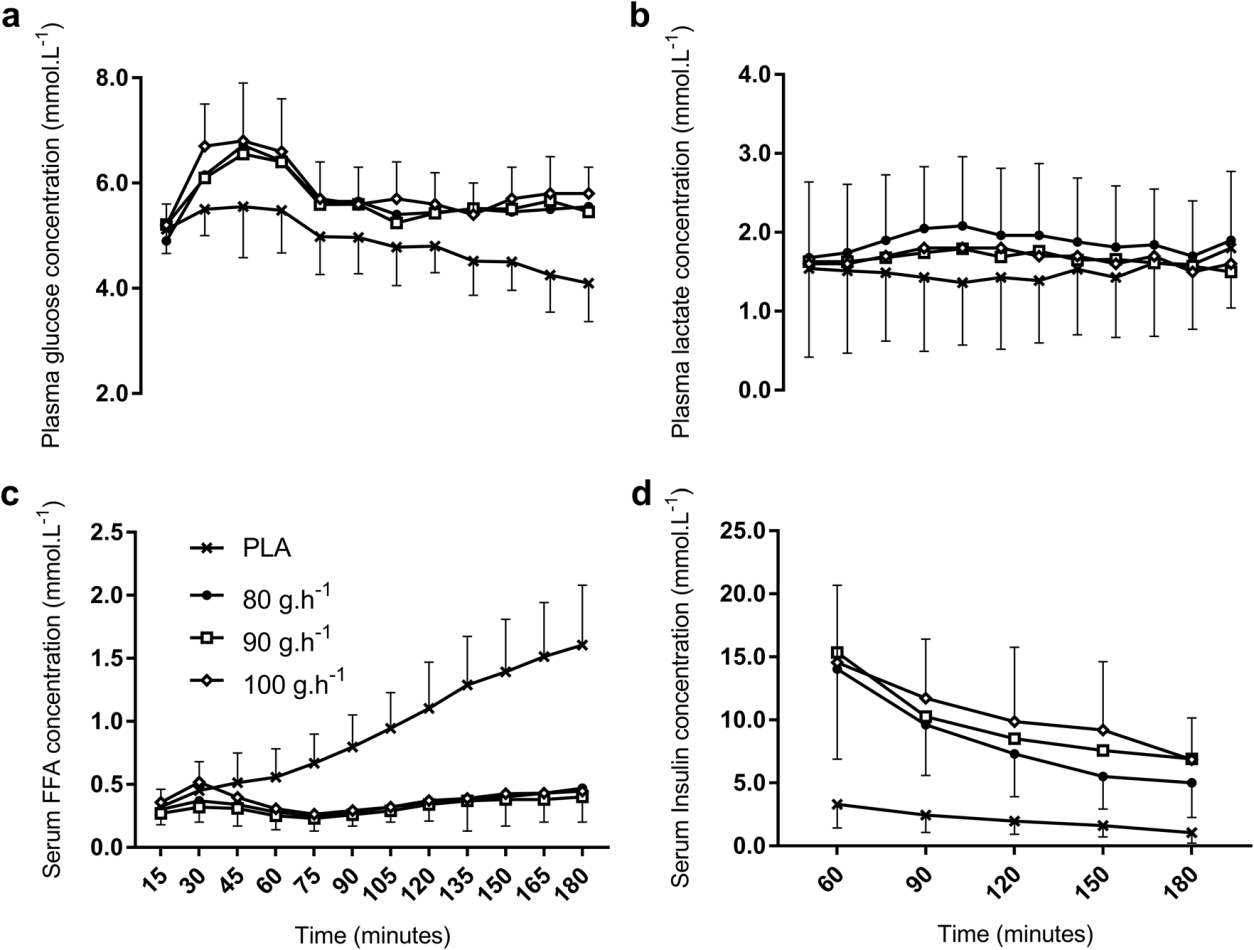
Circulatory metabolites, plasma glucose and lactate, serum-free fatty acids and insulin concentrations during the 3-h ride. See text for statistical and ES comparisons

Plasma glucose concentration increased at the onset of exercise with CHO ingestion, peaking at 6.8 ± 1.2 mmol L^−1^ with 100 g h^−1^, with only minimal differences between CHO conditions (ES < 0.17, *P* = 1.00, Fig. 4a). From 45 min onwards, there remained no differences between CHO conditions for plasma glucose during the entire 3-h ride. Plasma glucose was consistently lower in PLA than with CHO ingestion, with differences to all CHO conditions in the final hour significant and large (ES > 1.26, *P* < 0.045). Plasma lactate did not differ between conditions with mean concentrations during the 3-h ride of 1.5 ± 0.9 in PLA to 1.9 ± 0.8 with 80 g h^−1^ (ES < 0.4, *P* = 1.00, Fig. 4b). Serum-free fatty acid concentrations were increasing and significantly higher in PLA than with CHO ingestion from 60 min onwards, ES > 1.75, P < 0.05, Fig. 4c but were unchanged between CHO doses at all time points (ES < 0.8, *P* = 1.00). Serum insulin concentrations were significantly lower in PLA than with CHO ingestion in the 2nd and 3rd hours (ES > 1.8, *P* < 0.012, Fig. 4d) and declined during this period in PLA (2.3, 1.4 to 3.1 µU.L^−1^, ES = 1.48, *P* = 0.024). There were, however, no significant differences between CHO conditions in the 2nd and 3rd hours (ES < 0.58, *P* > 0.72) except at 150 and 180 min, where insulin concentrations were moderately, but not significantly, lower with 80 g h^−1^ than 90 and 100 g h^−1^ (ES = 0.71 and 0.81, *P* = 0.74 and 0.70, respectively).

### Time trial performance

**Fig. 5:**
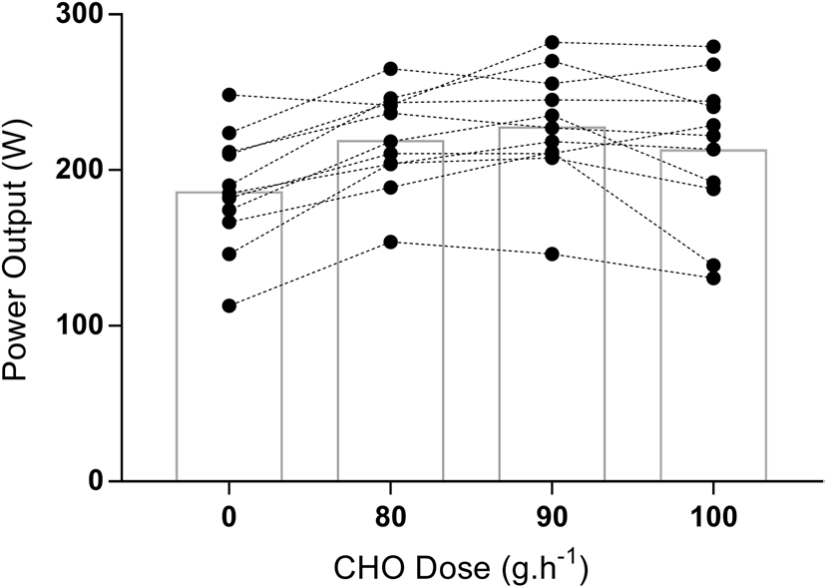
Power output during the 30-min time trial with individual performance data. See text for statistical and ES comparisons

Power output in the 30 min time trial was highest with 90 g h^−1^ (228 ± 37 W, Fig. 5). This was a 6.8% higher power output than 100 g h^−1^ (212 ± 48 W) and calculated to be a 93.0% likely/probable meaningful effect (14.8, − 0.4 to 30.1 W, ES = 0.40, *P* = 0.52 [Bonferroni]). Compared with 80 g h^−1^ (219 ± 32 W), this resulted in a 4.0% higher power output (8.8, − 0.6 to 18.3 W, ES = 0.26, *P* = 0.68), an 86.5% likely/probable meaningful effect. Against the placebo condition (186 ± 37 W), all 3 CHO doses produced a higher average power output (range 26.8 to 41.7 W, ES = 0.62 to 1.12, *P* = 0.012 to 0.001).

## Discussion

The data from the present study partially confirm our hypothesis that the ingestion rate of glucose–fructose solutions above previously reported intestinal saturation rates during 3 h of exercise negatively influenced subsequent time trial performance. This is likely explained by an increased reliance on muscle glycogen, rather than any real changes in exogenous CHO oxidation or glucose released from the liver. The effects were not statistically significant when multiple comparisons were accounted for but the observed effects were moderate and/or meaningful in the context of endurance exercise performance.

There were no observed changes in liver glycogen oxidation but a moderate (although non-significant) increase in muscle glycogen oxidation was seen when the dose of ingested CHO exceeded 90 g h^−1^, the primary findings of this investigation. Exogenous CHO oxidation increased when the CHO dose was marginally elevated above previously reported intestinal saturation rates for glucose and fructose, peaking at 1.60 g.min^−1^. In line with previous data, exogenous CHO ingestion spared fat oxidation and this was seen in all CHO doses (Smith et al 2019; Wallis et al. 2006). Finally, we observed that time trial performance following the 3-h prolonged ride was also superior with 90 g h^−1^, a novel finding with regard to exercise performance of this duration with exogenous CHO provision. Taken together, this suggests that the dose of ingested CHO should not exceed reported intestinal saturation rates as previously shown.

We previously reported that endurance exercise performance is diminished if rates of ingested CHO significantly exceed ≈ 60 g h^−1^ for glucose and 90 g h^−1^ for glucose–fructose mixtures (King et al. 2018). In the current study, exercise performance, measured by a 30-min time trial following a 3-h steady state ride at moderate intensity, was 3.7% and 7.5% higher with ingestion of 90 g h^−1^ than with 80 and 100 g h^−1^ respectively, suggesting a similar, albeit slightly smaller effect. Similar improvements have previously been reported, but largely only in comparison to placebo ingestion or isocaloric CHO strategies of differing composition (Angus et al. 2000; Currell and Jeukendrup 2008; Hulston et al. 2009; Madsen et al. 1996; Rowlands et al. 2008; Smith et al. 2010; Tripplett et al. 2010).

However, there are less data to draw upon when the total exercise duration is equal to or greater than 3 h. Conflicting data exist on the effect of CHO ingestion vs. placebo on 100 km TT performance, which in trained cyclists is of similar duration to the current study (Angus et al. 2000; Madsen et al. 1996). In these studies, both a beneficial effect (Angus et al. 2000) and no effect (Madsen et al. 1996) were observed when using similar doses of glucose (60 g h^−1^). In terms of CHO composition, Tripplett et al. (2010) reported improved 100 km TT time with glucose–fructose ingestion (108 g h^−1^) compared with an isocaloric dose of glucose only, supporting the need for multiple transportable CHO in prolonged (< 2.5 h) exercise. In the most comprehensive assessment of CHO dose and exercise performance to date, Smith et al. (2013) found the dose:performance relationship to be curvilinear, modelling an upper ingestion rate of 88 g h^−1^, which data from the current study support, where ingesting 100 g h^−1^ was detrimental. However, the possible physiological mechanisms supporting a CHO dose effect were not explored in these studies to which the present study provides new evidence. The improved performance in the current study also provides novel data to the sensitivity of metabolic responses to CHO doses that fall above and below the suggested ingestion rate of 90 g h^−1^ for prolonged exercise (Jeukendrup 2014).

It should be noted, however, that the magnitude of performance loss seen in the CHO doses above and below the best performance with 90 g h^−1^ in the current study did not reach statistical significance. However, with the often small margins of victory in endurance events, the observed improvement in power output in the 30-min TT (9–16W) was calculated to be likely/probable to be a meaningful effect. However, to detect meaningful changes in athletic performance, a move to enhance statistical analysis by including measures alongside traditional null hypothesis testing has been suggested (Bernards et al. 2017; Deighton et al. 2017). This study provides evidence that even small alterations in the ingested dose of CHO during exercise may impact subsequent time trial performance. This interpretation should be taken cautiously despite providing small to moderate effects, with 8 of 11 of the participants recording a superior power output with 90 g h^−1^. Translation to the greater population should be considered with some caution, but individual responses are important in terms of planning individualised nutrition strategies for endurance performance (Jeukendrup 2017).

Rates of exogenous CHO oxidation reached 1.60 g min^−1^ with 100 g h^−1^, although this was not significantly higher than the lower doses of 80 and 90 g h^−1^. However, all doses produced exogenous oxidation rates higher than 1 g min^−1^ due to the presence of fructose and non-competitive intestinal transport. Similarly, over the last hour of exercise, when CHO provision may be most impactful, the total amount of exogenous CHO utilised, namely by contracting muscle, was moderately higher with 100 g h^−1^ (though not significantly different) than both 80 and 90 g h^−1^. Such high exogenous CHO oxidation rates are comparable with the existing literature, but only with very high (135 g h^−1^ glucose–fructose) CHO ingestion rates (Jentjens et al. 2004a, b; Jentjens and Jeukendrup 2005). However, these studies did not report contributions of liver and muscle glycogen oxidation during (2 h) continuous cycling. Furthermore, it is also not possible to determine if the extremely high rates of exogenous CHO oxidation were beneficial to performance in these investigations, as no measure of performance was included. Data from our laboratory (King et al. 2018) support earlier work by Smith et al. (2013) suggesting that the very high doses used in those studies would actually be detrimental to endogenous fuel selection and performance. In the current study a small, but ‘likely/probable’ decrease in performance was seen despite a consistently moderate (~ 6–10%) higher rate of exogenous CHO oxidation with 100 g h^−1^.

Interestingly, the rate of exogenous CHO oxidation continued to rise throughout the 3-h ride. Jeukendrup et al. (2006) observed a similar rise until the same duration into exercise, when exogenous CHO oxidation plateaued during a 5-h exercise period with similar CHO ingestion (90 g h^−1^ glucose–fructose). Why exactly this rise occurs remains to be elucidated, but it is possible that in the early stages of exercise, or during shorter duration exercise, a portion of the ingested CHO is taken up by non-exercising peripheral tissues and not subsequently oxidised. It is known that glycogen synthase activity is directly upregulated by muscle contraction (Nielsen and Richter 2003) and in this phase of exercise this mechanism may serve to spare muscle glycogen before the prolonged demands of an exercise bout require activation of glycogen phosphorylase and reduction in glycogen synthase activity to enable the maintenance of exercise intensity. A limitation of the ^13^C tracer technique is the inability to differentiate any partitioning of ingested substrates, for which a dual tracer technique is required (Jeukendrup et al. 1999a, b). However, this effect may indicate that to some extent ingesting CHO during the early stages of exercise may provide a cumulative effect later in exercise when glycogen availability and oxidation are significantly lower, as observed in the current study.

The effect of exogenous CHO feeding on endogenous fuel utilisation has previously highlighted reductions in hepatic glucose production (Jeukendrup et al. 1999a, b) or liver glycogen oxidation (Wallis et al. 2006) as well as sparing of muscle glycogen (Tsintzas et al. 1996, 2001) as possible mechanisms to explain the ergogenic effect of CHO supplementation during endurance exercise. Furthermore, the role of multiple transportable CHO ingestion is now established to be beneficial over single (glucose or maltodextrin) source CHO for delivering higher rates of exogenous CHO oxidation and the potential to spare endogenous glycogen. However, the current study demonstrates for the first time that small alterations in the dose of ingested glucose–fructose modify endogenous fuel selection in line with small but meaningful (likely/probable) improvements in power output in a self-paced TT following 3 h of cycling. Based on previous research in similarly trained cyclists, we observed a significant and negative upregulation of total, liver and muscle CHO oxidation during 2 h of prolonged cycling when exogenous CHO delivery at 112.5 g h^−1^ exceeded intestinal absorption rates (King et al. 2018). Such an ‘over-dose’ effect appears to also diminish power output at the end stages of exercise, where in racing scenarios, the ability to perform at a higher exercise intensity is often required. Data from the current study suggest that a CHO ‘over-dose’ of lesser extent also provides a suboptimal modification of muscle glycogen utilisation but this effect also appears to be sensitive to the level of CHO consumed above intestinal saturation rates. The precise mechanism(s) explaining the loss of power output with CHO ingestion rates beyond the intestinal saturation levels has not yet been fully determined. However, exceeding doses of 90 g h^−1^ of glucose–fructose should be avoided in endurance exercise to mitigate the possible performance losses seen with increased reliance on muscle glycogen.

With the 90 g h^−1^ dose, a moderate reduction in the rate of muscle glycogen oxidation at 180 min was seen, supporting our previous findings (King et al. 2018). However, this was not statistically significant with multiple comparisons accounting for the ingestion of CHO above and below previously reported intestinal saturation limits. A limitation present in our study design is the lack of regulatory metabolic data that may provide further mechanistic detail at the muscle fibre in response to CHO feeding. With the observed increase in muscle glycogen oxidation, it stands that glycogen phosphorylase activity is increased, perhaps through CHO driven regulation of glycolysis. Indeed, a moderate reduction in the provision of fat to energy expenditure was also seen when the ingested CHO dose increased from 90 to 100 g h^−1^. This could perhaps be due to the slightly higher insulin concentrations during the 3rd hour (small ES at 120 and 150 min) suppressing adipose tissue FFA release, or through a non-insulin-dependent mechanism. Where fat oxidation is suppressed through insulin action and beta-oxidation-derived acetyl CoA is reduced, upregulation of PDH (and PKA) activity is increased to maintain energy requirements to the working muscle. Further, the action of adrenaline may stimulate glycogenolysis via β-adrenergic receptors and glycogen phosphorylase phosphorylation and glycogen synthase dephosphorylation by cAMP (Cohen 2002; Jensen and Richter 2012). However, future research should focus on the molecular and cellular modifications that exogenous CHO provision initiates.

## Conclusion

This study demonstrates that the ingestion of up to 90 g h^−1^ of carbohydrate (2:1 of glucose and fructose) is optimal for time trial performance following 3 h of sub-maximal exercise compared to higher doses and a non-CHO solution. However, there were only small alterations in substrate metabolism, with the exception of muscle glycogen. The moderately increased reliance on muscle glycogen observed with glucose–fructose ingestion above the previously reported intestinal saturation limits is a plausible explanation for the decreased performance at 100 g h^−1^. Supporting previous evidence that the CHO dose:performance relationship is not linear and that ‘over-dosing’ with exogenous CHO may be detrimental, the current study provides new evidence to answer the question of not achieving recommended CHO intakes of 90 g h^−1^ by small amounts. Here, our data suggest that the “over-dose” effect, whereby effective and beneficial muscle glycogen oxidation and exercise performance are diminished, is potentially sensitive to exceeding this target by as little as 10 g h^−1^. Therefore, it should be recommended that the rate of ingestion should reach, but not exceed intestinal saturation for glucose and fructose. Future research is warranted to confirm the precise mechanism at the muscle fibre level behind this effect in prolonged exercise.

## Abbreviations

CHO: Carbohydrate
CV: Coefficient of variation
ES: Effect size
GLUT5: Glucose transporter 5
HR: Heart rate
IRMS: Isotope ratio mass spectronomy
NMRS: Nuclear magnetic resonance spectroscopy
PLA: Placebo
SGLT1: Sodium-dependent glucose transporter 1
TT: Time trial
$\dot {V}{{\text{O}}_{2\;\hbox{max} }}$: Maximal oxygen uptake
